# Shun Bolted Flour—Dr. John Ellis Tells Why Americans Lose Their Teeth at an Early Age

**Published:** 1896-03

**Authors:** John Ellis

**Affiliations:** New York Record


### SELECTIONS.

### Shun Bolted Flour—Dr. John Ellis Tells Why Americans Lose Their Teeth at an Early Age.

BY JOHN ELLIS, M.D., IN NEW YORK RECORD.

What Dr. V. C. Bell is reported to have said in the *Recorder* of November 29th every careful observer can see to be correct, and, further, it is not simply the teeth of the rising generation which suffer, but also the bones, muscles, digestive organs and brain. Why is all this physical degeneration of our young people? We have not to look far for one of the chief causes. Many of our children are half starved, and some of them starved to death.

"Starved! Why, she eats enough!" exclaimed an astonished mother, when I told her that her young daughter was starving to death. There she lay helpless upon the bed, not able to turn herself, and with some symptoms of scurvy, but in good flesh. I quietly asked the mother what she ate. She replied: "She eats toast made from the very best superfine white flour. If she eats anything else she throws it up." I directed her mother to mix mashed potatoes with the flour from which she made her bread. She did so and the child recovered rapidly.

Careful experiments, made by Magendie and others, have demonstrated that animals can only live for a few weeks if fed only on superfine white flour, whereas, they can live and thrive on unbolted flour or meal without any trouble. The Lord intended the grain, as a whole, for human food, and He manifestly knew what he was doing when he created our cereals. The food required to nourish the teeth, bones, muscles, stomach, to enable it to properly digest our food, and the brain, is found in excess in the dark portion of the kernel which lies immediately beneath the hull, and the miller, in bolting, separates this portion as far as he can, and most of it is fed to cattle, horses, hogs, etc., and they have good teeth, muscles, stomachs and bones when thus fed.

The white portion of the kernel from which white flour is made contains an excess of starch, principally a heat and fat-producing material when taken as food, so that the whiter the flour the poorer it is. One simple fact ought to satisfy every intelligent man and woman that superfine white flour is not fit for human use, and that starvation must inevitably follow, to a greater or less extent, its use as food, viz., there is very little difficulty in keeping superfine white flour free from insects, must or mould, whereas it requires care and watchfulness to preserve unbolted flour and meal free from insects, must, etc. Do we want to feed our children upon a flour that will not sustain, for any considerable length of time, animal, insect or even vegetable life?

Dyspepsia is more prevalent in our country than, I think I can say, in any other. Superfine flour does not contain the nourishment required by the stomach to enable it to digest food. The prevalence of dyspepsia in our country and England has led a number of medical writers in England and in this country, of late, to condem the use of all cereals—wheat, rye, oats, etc.—as food, claiming that the starch overtaxes the stomach, and that we should use as food nothing but nuts and fruits, and if we find them not sufficient we should use a little meal or animal food, they think. But if we use the dark or coarse portion of the grain as well as the white, the stomach will be nourished, the whole grain will be digested, and it will not cause dyspepsia.

In cases of irritable or weak stomachs from the use of superfine flour, it will be well to sift out the coarsest of the bran for a time, until the stomach gains strength. Cases of dyspepsia have been cured by simply boiling the wheat for a few hours and then eating, it chewing it carefully. Banish superfine flour, and bread and cakes made from it from our land, or from use in our households, and there would be a wonderful change for the better in the development of the young, not only as to their teeth, but also as to all the structures of the body. No parent who cares for the development, health and comfort of his or her children should, in my estimation, ever allow a single pound of superfine flour, or bread or cakes made from such flour, to enter his or her house.

Having constantly in view the development and health of our race, I have traveled over our own country from the East to Alaska and California in the West, and Florida in the South, over most of the countries of Europe, Egypt and Western Asia, and I can say, as a result of my observation, that wherever the people eat, instead of superfine flour, the meal or flour of the whole grain, be it wheat, rye, oats or barley, they have good teeth, are well developed, and are rarely troubled with dyspepsia. For more than forty years I have carefully avoided the use of superfine flour, stimulants, narcotics and condiments, excepting sugar and salt, and although my eightieth birthday passed two days ago, I rarely, if ever, fail to have a good appetite, and my food tastes as well as it did when I was a boy, and I have more than half of my teeth left.

---

SIXTY-FIVE great trunk lines of railway have laws forbidding their employes to enter saloons or drink spirits of any kind while on duty. Ten of these roads have rules discharging without question any of their employes who frequent saloons.

